# The French National Registry of patients with Facioscapulohumeral muscular dystrophy

**DOI:** 10.1186/s13023-018-0960-x

**Published:** 2018-12-04

**Authors:** Céline Guien, Gaëlle Blandin, Pauline Lahaut, Benoît Sanson, Katia Nehal, Sitraka Rabarimeriarijaona, Rafaëlle Bernard, Nicolas Lévy, Sabrina Sacconi, Christophe Béroud

**Affiliations:** 1 Aix Marseille Univ, INSERM, MMG, Bioinformatics & Genetics, Marseille, France; 2 Université Côte d’Azur, Service Système Nerveux Périphérique, Muscle et SLA, Centre Hospitalier Universitaire de Nice, Nice, France; 3 APHM, Hôpital Timone Enfants, Laboratoire de Génétique Moléculaire, Marseille, France; 4 Institute for Research on Cancer and Aging of Nice (IRCAN), INSERM U1081, CNRS UMR 7284, Université Côte d’Azur (UCA), Faculté de Médecine, Nice, France

**Keywords:** Patient registry, Database, Facioscapulohumeral muscular dystrophy, FSHD, FSHD1, FSHD2, Myopathy

## Abstract

**Background:**

Facioscapulohumeral muscular dystrophy is a rare inherited neuromuscular disease with an estimated prevalence of 1/20,000 and France therefore harbors about 3000 FSHD patients. With research progress and the development of targeted therapies, patients’ identification through registries can facilitate and improve recruitment in clinical trials and studies.

**Results:**

The French National Registry of FSHD patients was designed as a mixed model registry involving both patients and physicians, through self-report and clinical evaluation questionnaires respectively, to collect molecular and clinical data. Because of the limited number of patients, data quality is a major goal of the registry and various automatic data control features have been implemented in the bioinformatics system. In parallel, data are manually validated by molecular and clinical curators. Since its creation in 2013, data from 638 FSHD patients have been collected, representing about 21% of the French FSHD population. The mixed model strategy allowed to collect 59.1% of data from both patients and clinicians; 26 and 14.9% from respectively patients and clinicians only. With the identification of the FSHD1 and FSHD2 forms, specific questionnaires have been designed. Though FSHD2 patients are progressively included, FSHD1 patients still account for the majority (94.9%). The registry is compatible with the FAIR principles as data are Findable, Accessible and Interoperable. We thus used molecular standards and standardized clinical terms used by the FILNEMUS French network of reference centers for the diagnosis and follow-up of patients suffering from a rare neuromuscular disease. The implemented clinical terms mostly map to dictionaries and terminology systems such as SNOMED-CT (75% of terms), CTV3 (61.7%) and NCIt (53.3%). Because of the sensitive nature of data, they are not directly reusable and can only be accessed as aggregated data after evaluation and approval by the registry oversight committee.

**Conclusions:**

The French National Registry of FSHD patients belongs to a national effort to develop databases, which should now interact with other initiatives to build a European and/or an international FSHD virtual registry for the benefits of patients. It is accessible at www.fshd.fr and various useful information, links, and documents, including a video, are available for patients and professionals.

**Electronic supplementary material:**

The online version of this article (10.1186/s13023-018-0960-x) contains supplementary material, which is available to authorized users.

## Background

With an estimated prevalence between 1/20,000 and 1/8,000 [1, 2], autosomal dominant Facioscapulohumeral muscular dystrophy (FSHD) is globally the third most common inherited myopathy, while, in adult, it is the second most frequent myopathy after the Steinert Myotonic Dystrophy (DM1).

FSHD is characterized by an important clinical variability including age of onset, disease progression, and presence or absence of multisystemic involvement [3]. FSHD patients are typically characterized by a progressive and asymmetric involvement of facial, shoulder girdle, anterior forelegs and abdominal muscles. Pelvic and axial muscles can also be affected. The disease progression is usually very slow and only 10–20% of patients with FSHD will become wheelchair-bound after age 50 [4]. This portion increases to more than 40% by age 18 in the early form of the disease [5]. Respiratory involvement is infrequent and, in the majority of cases, has been reported in wheelchair-bound patients or as secondary to the skeletal deformation (cyphosis, hyperlordosis) that can be seen in severe forms of the disease [6]. Cardiac involvement, manifesting as a predilection to atrial arrhythmias, is seen in about 5% of patients, few of whom require treatment [7]. Sensorineural deafness has been described in a variable percentage of FSHD patients, ranging from 25 to 65% in different studies [8–11]. Retinal vascular involvement is rare and is frequently presented as asymptomatic retinal telangiectasia. Nevertheless, in severely affected FSHD patients, retinal abnormalities can have dramatic consequences including complete loss of vision due to retinal exudation leading to retinal detachment (Coat’s syndrome) [12–14]. Dysphagia has been reported as affecting 10% of adult FSHD patients and has been interpreted as secondary to the involvement of orofacial muscles [15]. In childhood-onset FSHD patients, tongue atrophy, epilepsy and mental retardation have been described as an uncommon feature [16]. Other symptoms frequently reported by FSHD patients are pain and fatigue [17, 18]. Differences in expression of this disease related to gender have been described, women tending to be less symptomatic than men (late disease onset and slower progression) [19, 20]. Life expectancy is not modified except for patients with a childhood-onset FSHD.

FSHD is a genetically heterogeneous disease with two distinct forms. FSHD type 1 (FSHD1) is the most common form with more than 90% of FSHD patients, while the FSHD type 2 (FSHD2) is the rarest form of the disease, affecting less than 5% of FSHD patients. FSHD1 is caused by the contraction to 10 repeats or less of the 3.3 Kb D4Z4 repeat units on chromosome 4. In contrast, FSHD2 is caused by mutations in the chromatin modifier *SMCHD1* gene located on chromosome 18. Alternatively, pathogenic mutations in the *DNMT3B* gene have been described in association with FSHD2 [21].

To date, in France, molecular diagnosis is available only for the FSHD1 type; indeed, clinical FSHD patients with no D4Z4 contraction might be explored in a research context, and positive results are not fully integrated in the diagnosis process yet.

With the recent interest from pharmaceutical industries to develop novel therapeutic solutions for FSHD patients, it is now mandatory to create national registries to facilitate fully characterized patients’ recruitment in clinical trials and better understand the natural history of the disease to identify key parameters to efficiently evaluate treatment benefits. Today various national registries have been or are being set up as reported in the TREAT-NMD website (http://www.treat-nmd.eu). They are either disease-centric in the United-Kingdom [22], Italy [23], Czech Republic, Netherlands, or part of larger registries including multiple neuromuscular diseases in the United States [24], Canada [25], Egypt, New-Zealand, Spain and Russia. A recent ENMC international workshop was dedicated to FSHD registries to ultimately harmonize data content to allow data sharing and match-making between registries [26]. Here we report the creation of the French national FSHD registry and highlight its original design allowing a strong involvement of both patients and physicians, and its evolution since 2013.

## Materials and methods

### Database structure

The FSHD French National Registry is accessible at www.fshd.fr. It is composed of a secured-access database for registered users, and a freely accessible website for the general public. The database was built as a relational database (45 tables, 44 inter-table links and 533 fields) with the 4th dimension package (4D company, http://www.4d.com/). The website contains a mix of static and dynamic pages created upon request, using JavaScript and Ajax functions (https://www.w3schools.com/). The web interface was developed using the HTML5 and CSS3 standards with the Bootstrap 3.1 package, thus allowing easy access to the website from multiple platforms (computers, tablets or smartphones).

The database structure is modular allowing the addition of any field or feature. It contains both clinical and molecular data from FSHD patients collected via either self-report questionnaires, or clinical evaluation questionnaires filled by physicians, or both:

- The self-report questionnaire collects general data, a clinical self-assessment, genetic diagnosis results, and the patient’s disease history (Additional file 1: Figure S1).
- The clinical evaluation questionnaire collects general data, diagnosis results, the patient’s medical history, and a full clinical evaluation including a manual muscular testing (Additional file 2: Figure S2 and Additional file 3: Figure S3).

The database is accessible to three different categories of users: 1) the patients who can fill self-report questionnaires and visualize both self-report and clinical evaluation questionnaires; 2) the physicians who can access their patients’ data and 3) the clinical and molecular curators who ensure the high quality of data stored in the database through a manual quality control. For each patient, multiple self-report and clinical evaluation questionnaires can be created, generally every eighteen months. Self-report questionnaires can only be filled by patients with FSHD1, while two versions of the clinical evaluation questionnaire are proposed, one for FSHD1 and one for FSHD2 patients.

### Data security

The legal approval for the French FSHD registry has been obtained from the CNIL (French National Committee for Informatics and Liberty) under authorization No. 912291 on June 25th, 2012. The CCTIRS (French advisory committee on information treatment for research in health) also gave a favorable opinion No. 12.004bis. Furthermore, a written informed consent was obtained from every patient before entering any data into the registry.

Web access to the registry is secured by a two-factor authentication process combining a login and a password. An additional layer of security was added through the use of a session-specific token. Supplemental security measures have also been implemented, including login throttling and inactivity timeout.

Daily backup and a log file are used to avoid loss of data in case of system failure.

### Data collection: A mixed model involving patients and physicians

The French National Registry of patients with FSHD has been developed to collect clinical and molecular data obtained from two sources: a self-report questionnaire filled by the patient and a clinical evaluation questionnaire filled by both the patient and the physician providing follow-up care. The questionnaires are sent to the clinical curator who controls the data quality prior to their import into the database. Once the patient has been created, a unique Personal Identification Number (PIN) code is sent to the patient and to the physician if a clinical evaluation form was filled.

**Fig. 1:**
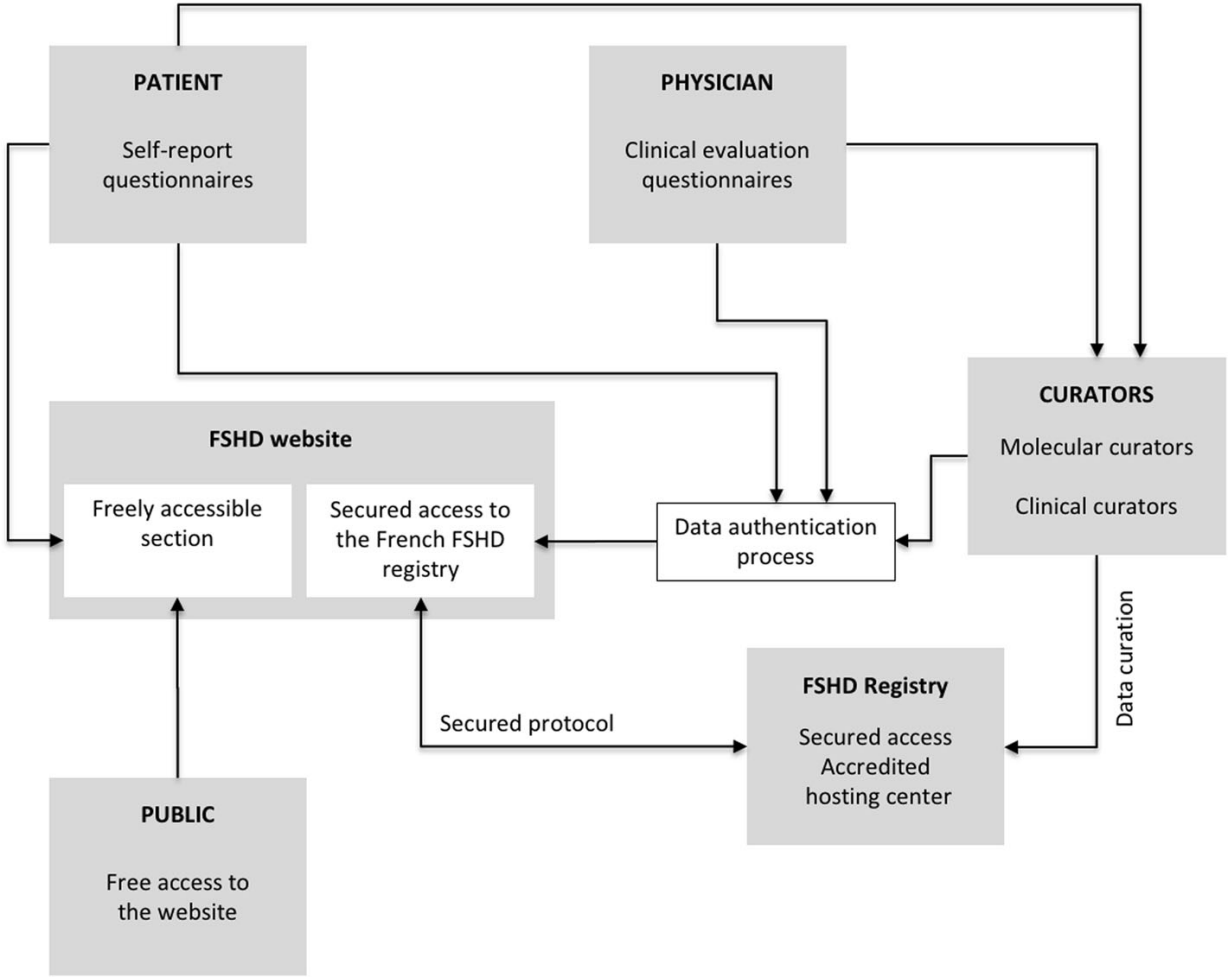
Actors of the French National Registry of FSHD patients

As patients are followed within a highly structured national network of reference centers, physicians can be granted access to their patients’ data only or to patients registered in their reference center. In parallel, to simplify the patient’s mobility within the network of reference centers, each patient is affiliated to a specific group and can be moved from one center to another. Actors and their interplay are illustrated in Fig. 1.

### Clinical and molecular data validation

Two levels of data curation are available: the clinical curator can create new patients and new self-reports or clinical evaluation questionnaires, while the molecular curator can only fill data in existing questionnaires. Both curators have access only to data related to their domain of expertise, i.e. clinics and genetics. A questionnaire is considered validated only when the two sections are validated. All mandatory items thus have to be filled in (Additional file 1: Figure S1, Additional file 2 and Additional file 3). To simplify the data collection and validation process, a color code allows the rapid identification of missing data. In parallel, the curator can duplicate similar data when completing the patients’ follow-up questionnaires.

### Data quality

Various features have been implemented to ensure a high data quality. They allow the following automatic actions:

- *Data pre-filling based on previous responses in the questionnaire.* For instance, if the curator checks “No pain” to the “*Pain area*” question, then the answer “No” is automatically applied to the question “*Muscular and joint pain*” and the item “*Average daily pain*” is set to “0.0” (Additional file 4: Table S1).
- *Identification of potential conflicts*. If inconsistency is detected between various answers, an alert notifies the curator. For instance, if the curator checks “No” to the question “*Ambulation*” and then “Done” to the question “*10MT walking test*”, an alert is generated (see Additional file 5: Table S2 for set of consistencies rules).
- *Identification of patient duplicates*. When creating a patient, the curator is automatically notified if this patient already exists in the database even he is affiliated to a different reference center.

As previously mentioned, inconsistent or missing data are highlighted on the interface. Nevertheless, because questionnaires contain multiple pages, they can be filled through a multi-step process. To simplify data collection, the percentage of completion is displayed during the filling process. Additionally, an automatic summary form of missing data can be generated at any stage of the data entry process. The clinical curator can send the summary form with additional notes to the physician to collect missing data.

### Data analysis functionalities

Various graphical displays and tables have been designed to facilitate data analysis. The first set is dedicated to database evolution (number of patients and questionnaires) and data quality (with an emphasis on missing data). The second set extracts gender- and age-related data. The third set displays the medical specialties of the physicians who include patients in the registry. The fourth set contains data related to the molecular diagnosis: number of patients per laboratory and diagnosis results. The last one provides a graphical display of the patients’ population based on selected clinical criteria.

## Results

### Description of the FSHD French National Registry cohort

**Fig. 2:**
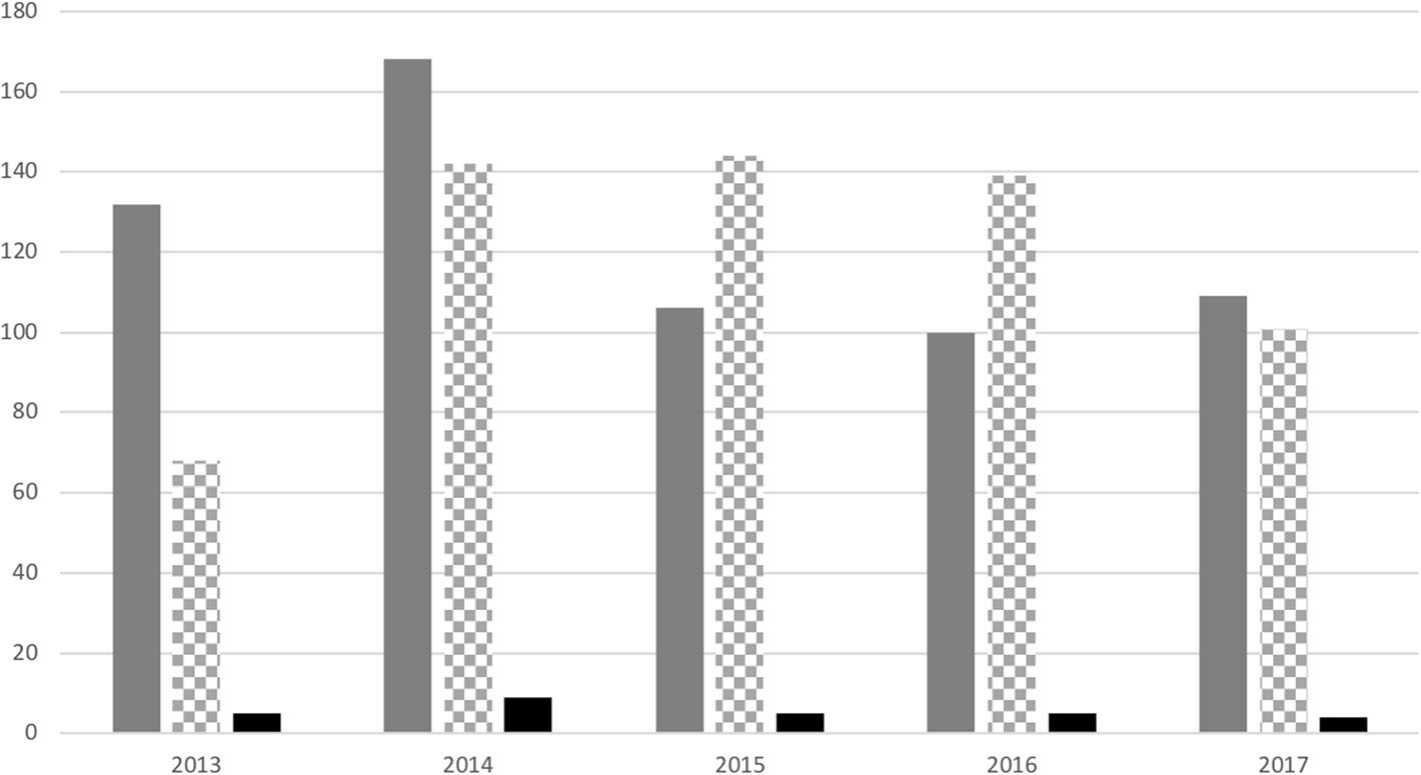
Data collection between 2013 and 2017. Number of patients with data collected through self-report questionnaires (plain grey), and clinical evaluation questionnaires for FSHD1 patients (checkerboard pattern) and FSHD2 patients (plain black)

The FSHD French National Registry was approved in June 2012 and set up in 2013. Considering the FSHD prevalence (1/20,000) and the French population (65 millions), we can estimate that about 3000 FSHD patients are living in France. During the last 5 years, we collected clinical and molecular data from 638 FSHD patients over the country representing about 21% of the French FSHD population. Data from 26% of patients were collected only from self-report questionnaires; 14.9% from a clinical evaluation questionnaire; while for the remaining 59.1% of patients, data were collected from both questionnaires (Fig. 2).

The FSHD1 form of the disease currently accounts for 94.9% of all clinical evaluation questionnaires. Improving the collection of data from FSHD2 patients is of paramount importance to gain insight into the natural history of the disease. On average, there are 97% of mandatory items completed for the self-report questionnaires and 88.4% for the clinical evaluation questionnaires.

**Fig. 3:**
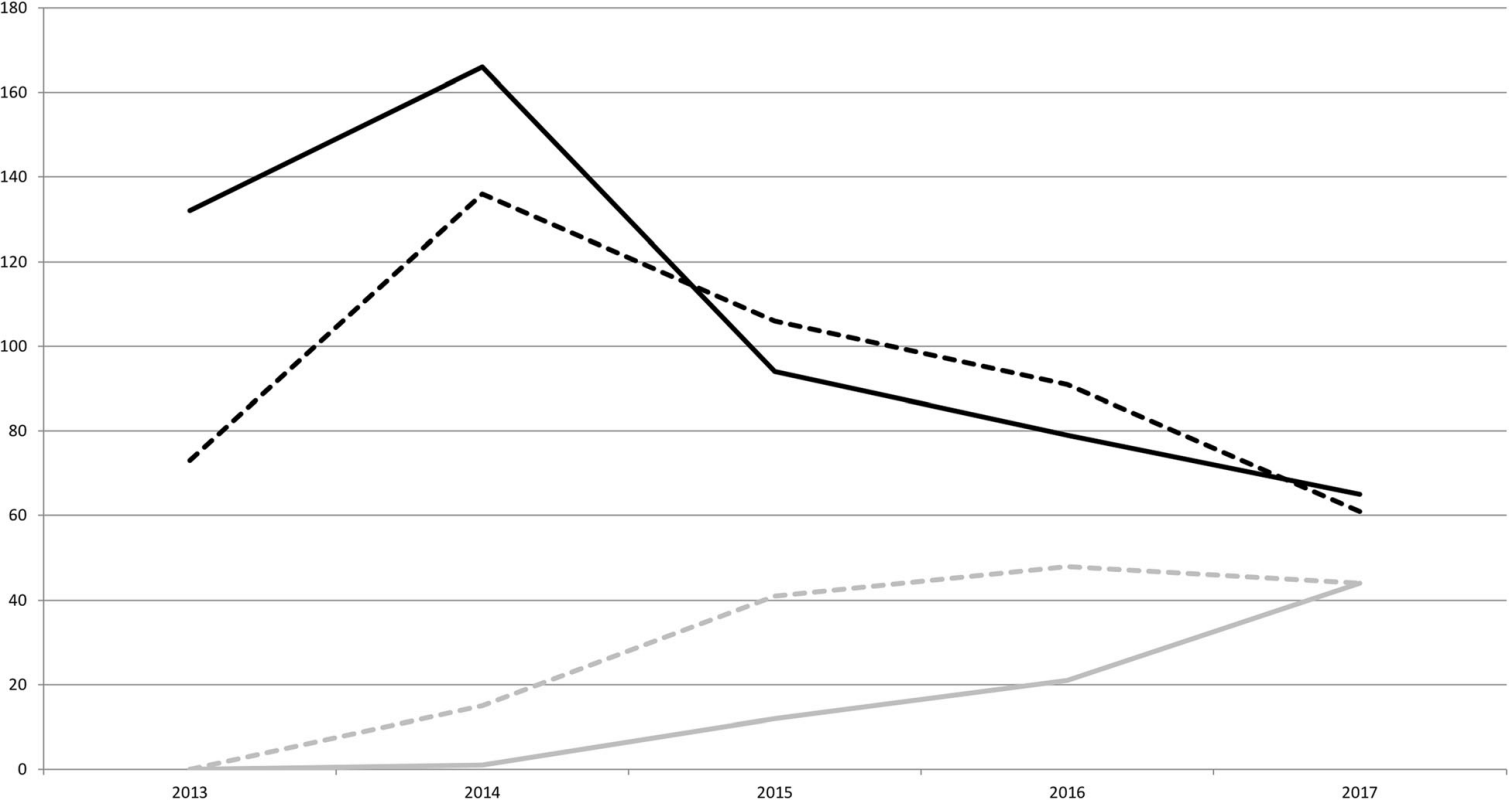
Number of inclusion and follow-up questionnaires between 2013 and 2017. Black = inclusions; Grey = follow-up; Plain lines = self-report questionnaires; dashed lines = clinical evaluation questionnaires

Over the five-years period of enrolment, follow-up questionnaires have been gleaned for registered patients at an increasing rate. They include 148 clinical evaluations and 78 self-report questionnaires (Fig. 3). This enrolment level has been reached thanks to the active participation of patients and neurologists located in more than 20 sites (Additional file 6: Figure S4). Patients were also included in the French overseas departments “Réunion” and “Martinique”.

**Fig. 4:**
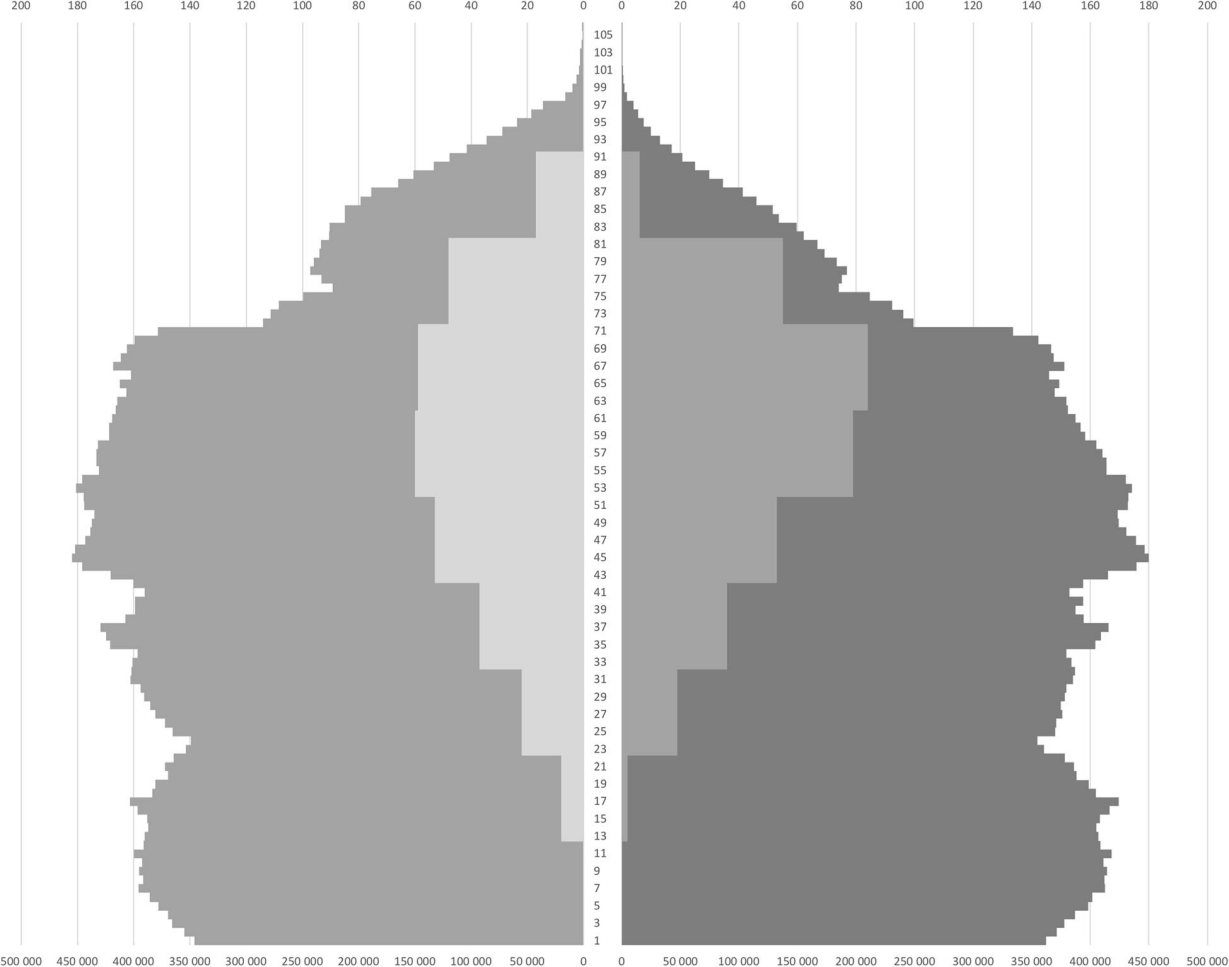
Age composition of the French FSHD registry population. Left = women; right = men. Dark grey = French population; light grey = FSHD population. Bottom Y-axis = number of individuals in the French population by age; Top Y-axis = number of FSHD patients by age group (every 10 years)

As shown in Fig. 4, the FSHD population sampled in the registry shows an under-representation of children and young adults. This is in agreement with the late age of onset of the disease [5]. Indeed, only a small proportion of FSHD patients display an early form of the disease. In addition, as the disease is slowly progressive, young adults are unregularly followed, in contrast to elders. Note that older FSHD patients, which are the most affected individuals, are strongly represented in the registry, underlining the limited impact of the disease on life expectancy. A difference in the age distribution is observed between men (52.6%) and women (47.4%), with women displaying a slightly broader age range than men.

### Disease severity

As reported by many authors [23, 27, 28], the severity of FSHD is highly variable. To better understand the variability of the pathology and be able to select homogeneous groups of patients eligible for future clinical trials, we included a detailed clinical description of each patient (Additional file 1: Figure S1, Additional file 2: Figure S2 and Additional file 3: Figure S3). To ensure interoperability with other registries, we mapped these clinical features to existing ontologies, nomenclatures and dictionaries. We selected reference systems including: the Clinical Trials Ontology (CTO), the Read Codes Clinical Terms Version 3 (CTV3), the Human Phenotype Ontology (HPO), the International Classification of External Causes of Injuries (ICECI), the International Classification of Functioning, Disability and Health (ICF), the Logical Observation Identifier Names and Codes (LOINC), the Medical Dictionary for Regulatory Activities (MEDDRA), the Medical Subject Headings (MESH), the National Cancer Institute Thesaurus (NCIt), the NIH NLM Value Sets (NLMVS), the Online Mendelian Inheritance in Man (OMIM), the PhenX Phenotypic Terms (PHENX), the SMASH Ontology (SMASH), the Systematized Nomenclature of Medicine - Clinical Terms (SNOMEDCT) and the Experimental Conditions Ontology (XCO). As shown in Additional file 7: Table S3, we were able to map more than 50% of the 60 features in only 3 systems: 45 (75%) in SNOMED-CT, 37 (61.7%) in CTV3 and 32 (53.3%) in NCIt. Only a limited number of features was directly mapped to HPO.

**Fig. 5:**
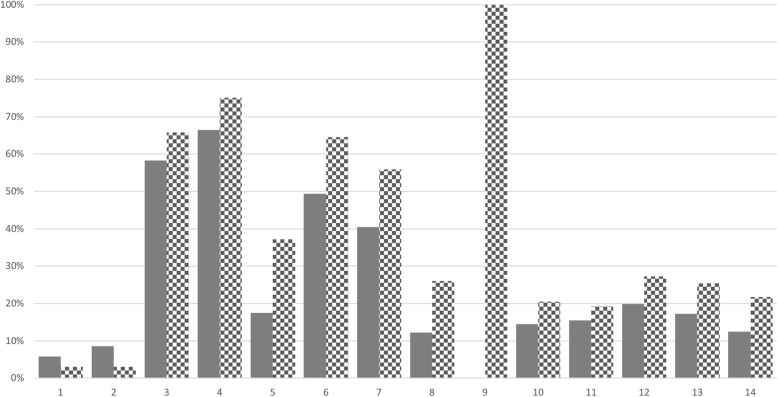
Associated clinical features for FSHD patients without (grey) or with (checkerboard pattern) respiratory involvement. 1 = Dry eyes; 2 = Inability to fully close eyes during sleep; 3 = Eyelid closure; 4 = Pucker: able to whistle / mimic a kiss; 5 = Wrinkle forehead; 6 = Smile; 7 = Show teeth; 8 = Cardiac involvement; 9 = Respiratory involvement; 10 = Gastrointestinal involvement; 11 = Hearing involvement; 12 = Ocular involvement; 13 = Metabolic involvement; 14 = Endocrine involvement

The French FSHD registry contains various analysis tools to rapidly evaluate complex clinical features. By selecting one or multiple features, the user can see how many patients harbor a given clinical description in combination with other features. For example, the comparison of patients with or without a functional respiratory involvement is displayed in Fig. 5. Patients displaying a respiratory involvement have a higher disease severity with two times more heart and endocrine involvements. In contrast, they have two times less inability to wrinkle forehead and to have dry eyes, and three times less inability to fully close eyes when asleep.

## Discussion

From an international perspective, the French National Registry of patients with FSHD is one of the 13 FSHD registries set up by the end of 2016 [26]. The French registry was designed to ensure the active participation of both patients and professionals (physicians and geneticists). To this end, we use a data-collection mixed model combining self-report and clinical evaluation questionnaires, filled by patients and professionals respectively. While this approach has already been successfully adopted for other Neuromuscular registries [29], such a dual approach is rare among existing FSHD registries at the international level as, to our knowledge, only the UK registry has adopted this approach [22]. Most other registries collect data via forms filled by medical practitioners, while the US registry has a self-report component, but its scope is essentially restricted to patient recruitment and expanding knowledge on the burden of the disease [30]. Besides, some FSHD registries are part of larger projects aiming at collecting data from major neuromuscular pathologies [24, 25]. In the French FSHD registry, most items in the clinical evaluation form are mirrored in the self-report questionnaire to assess data quality and identify, for each item, who is best suited to provide the answer (either the patient or the physician).

The French registry includes a strong patient-centered component. Indeed, the self-administered questionnaire has been designed by patients from the “groupe FSH”, a satellite association from the AFM. The questionnaire design was part of a study sponsored by the Nice University Hospital (10-AOI-07).

The French FSHD registry includes various tools to visualize and analyze data. For instance, it is possible to display the number of concomitant affections among patients suffering or not from a given affection as illustrated in Fig. 5 for a respiratory affection. Data from the French FSHD registry will thus allow assessing the nature and prevalence of comorbidities.

Because this registry contains sensitive data, it was approved by the appropriate French review boards (CNIL and CCTIRS) and is hosted within a secured environment. The registry is accessible through a website (http://www.fshd.fr) containing the secured database itself with a freely accessible part. It contains general information on the French National Registry, the disease, the diagnosis, the associated French network centers, the state of research, reference publications and clinical trials. Links to other national registries are provided. Video and various documents are available and can be downloaded and used to join the registry. Newsletters also ensure regular information to patients about the registry evolution and advertise about forthcoming regional meetings with experts.

To help achieve a high data quality level required for a fruitful exploitation of the registry, we developed computer assisted data input. Various data consistency and data pre-filling rules have thus been defined to assist the curator (Additional file 4: Table S1, Additional file 5: Table S2).

After 5 years, the registry now contains about 21% of French FSHD patients. The proportion of registered FSHD patients is expected to be further increased through the strong support provided by the French association dedicated to Muscular Dystrophies (AFM-Telethon) and the active participation of experts from the French national network for rare neuromuscular diseases (FILNEMUS), which is one of the 23 rare disease healthcare networks built through the second French national plan on rare diseases.

To ensure interoperability at the global level, we use genetics standards coupled to clinical description. We therefore mapped these terms to ontologies, dictionaries and terminology systems to evaluate if one of the tested systems can be used for match-making [31]. We observed that no system can be used to fully describe patients clinical features: the match rates of SNOMED-CT, CTV3 and NCIt are respectively of 75, 61.7 and 53.3%. Other ontologies and dictionaries, including HPO, contain less than 50% of terms used in the database. This underlines that interoperability between databases and registries cannot be ensured by a single approach and that systems must be able to handle heterogeneous queries to ensure efficient interoperability. While the rare disease field is trying to develop the Human Phenotype Ontology (HPO) and the Orphanet Rare Disease Ontology (ORDO) to simplify interoperability, our experience demonstrates that HPO still needs improvements to allow direct mapping of used clinical descriptions. In fact, the systems that showed the highest matching rates in the mapping process are not ontologies but a clinical health terminology system (SNOMED-CT), a clinical term system (CTV3) and a reference terminology system (NCIt). Future developments made through international initiatives as RD-Connect [31], the ELIXIR [32], the IRDiRC [33] and others, should ensure the inclusion of these terms and concepts in HPO. Meanwhile, match-making and other initiatives to link rare disease resources should consider using multiple ontologies, dictionaries and terminology systems.

## Conclusions

The French National Registry of FSHD patients is part of a national effort to develop national databases [22–25]. These initiatives should now interact with other initiatives to build a European and/or an international FSHD virtual registry. The French registry is applying the FAIR principles as data are Findable, Accessible, Interoperable and Reusable. While individual data are protected, collaborators can have access to aggregated data after their requests have been granted by the registry oversight committee. This data sharing policy will evolve in the future with the emergence of the European Reference Networks (ERN) and the European Joint Program (EJP) on rare diseases and the associated actions to promote data standardization and interoperability.

FSHD patients will benefit of the development and improvement of the French National FSHD Registry. Thanks to collected data, the registry will facilitate and accelerate clinical and translational research on the disease. Furthermore, specific guidelines on FSHD medical care will be regularly updated and disseminated, contributing to the national standardization of medical care for FSHD patients. Moreover, thanks to the registry, the assessment of feasibility and recruitment in clinical trials and studies will be facilitated. Despite strong efforts from physicians to ensure the collection of longitudinal data, this aspect is always a weakness in patients’ registries. In order to make it more efficient, we are currently modifying the system to allow the direct data submission from patients. The curators will then validate these new information to ensure their quality.

The regular update of the website will contribute to the improvement of the knowledge of both patients and professionals on FSHD, as well as facilitate interaction with academic and industrial partners. In addition, through the website, users already have access to educational links and other relevant resources (for example FSHD meetings and congresses).

## Additional files

Additional file 1: **Figure S1**. French National FSHD registry - SELF REPORT FORM (PDF 1194 kb)

Additional file 2: **Figure S2**. French National FSHD registry - INCLUSION FORM (PDF 431 kb)

Additional file 3: **Figure S3.** French National FSHD registry - INCLUSION FORM – *FSHD LIKE (PDF 473 kb)*

Additional file 4: **Table S1.** Pre-filling rules for the self-report and the clinical evaluation questionnaires. (PDF 59 kb)

Additional file 5: **Table S2.** Conflicts identification rules for the clinical evaluation questionnaire. (PDF 54 kb)

Additional file 6: **Figure S4.** Network of reference centers contributing to the French FSHD registry (PNG 112 kb)

Additional file 7: **Table S3**. Mapping of clinical terms to reference systems. CTO = the Clinical Trials Ontology; CTV3 = the Read Codes Clinical Terms Version 3; HPO = the Human Phenotype Ontology; ICECI = the International Classification of External Causes of Injuries; ICF = the International Classification of Functioning, Disability and Health; LOINC = the Logical Observation Identifier Names and Codes; MEDDRA = the Medical Dictionary for Regulatory Activities; MESH = the Medical Subject Headings; NCIt = the National Cancer Institute Thesaurus; NLMVS = the NIH NLM Value Sets; OMIM = the Online Mendelian Inheritance in Man; PHENX = the PhenX Phenotypic Terms; SMASH = the SMASH Ontology; SNOMED-CT = the Systematized Nomenclature of Medicine - Clinical Terms; XCO = the Experimental Conditions Ontology. (PDF 40 kb)

## Abbreviations

AFM-Telethon: French association dedicated to muscular dystrophies
CCTIRS: French advisory committee on information treatment for research in health
CNIL: French national committee for informatics and liberty
CTO: Clinical trials ontology
CTV3: Read codes clinical terms version 3
DM1: Myotonic dystrophy
EJP: European joint program
ERN: European reference networks
FILNEMUS: French national network for rare neuromuscular diseases
FSHD: Facioscapulohumeral muscular dystrophy
FSHD1: FSHD type 1
FSHD2: FSHD type 2
HPO: Human phenotype ontology
ICECI: International classification of external causes of injuries
ICF: International classification of functioning, disability and health
IRDiRC: International rare diseases research consortium
LOINC: Logical observation identifier names and codes
MEDDRA: Medical dictionary for regulatory activities
MESH: Medical subject headings
NCIt: National cancer institute thesaurus
NLMVS: NIH NLM value sets
OMIM: Online mendelian inheritance in man
ORDO: Orphanet rare disease ontology
PHENX: PhenX phenotypic terms
PIN: Personal identification number
SMASH: SMASH ontology
SNOMEDCT: Systematized nomenclature of medicine - clinical terms
XCO: Experimental conditions ontology
