# Supplementary material for: The French National Registry of patients with Facioscapulohumeral muscular dystrophy
Source: Orphanet J Rare Dis. 2018 Dec 4;13:218. doi: 10.1186/s13023-018-0960-x (PMC6280451; doi:10.1186/s13023-018-0960-x)
Supplement: Supplementary file 1 — Figure S1. French National FSHD registry - SELF REPORT FORM (PDF 1194 kb) [file 13023_2018_960_MOESM1_ESM.pdf]

French National FacioScapuloHumeral Muscular Dystrophy Patient Registry – *SELF-REPORT QUESTIONNAIRE*

- **PLEASE READ THE INSTRUCTIONS** provided with this form. They provide explanations for each question, **especially for the questions indicated by this sign**: ⚠
  - **Answer all « mandatory » questions: otherwise you won't be included in the registry.**
  - **Answer as many « highly encouraged » questions as you can.** If you make a mistake, please make sure that your corrections are understandable.
  - **If you have any questions or doubts**, you can ask your referring doctor.

→ ***This is the first time I fill this questionnaire***: ☐ **Yes** ☐ **No → *If No*:** ☐ **PIN code: __ __ __ __ __** ☐ ***I forgot my PIN code***

Thanks to the PIN code, you can access your data on the website: www.fshd.fr

This PIN code will be given to you by the doctor managing your FSHD (indicated in question #2) during your next visit.

Completion date: __ __ / __ __ /20__ __

MANDATORY QUESTIONS - *If you don't answer the mandatory questions, we won't be able to include your details in the FSHD registry (except for the "optional" questions)*

1. YOUR PERSONAL DETAILS

Last name *(first letter)*: ___ **First name** *(first letter)*: ___ **Maiden name** (*first letter*): ___ **Gender**: F ❑ M ❑

Date of birth: |___|___| / |___|___ |___|___| (month / year) City of residence: .... and/or Postal code: ____ ___ ___ ___ ___

2. What are the name and address of the doctor managing your FSHD? (very important question)

Pr/Dr *(Last name, First name)* .... Phone number *(**optional**)*: ....

Address: ....

3. Your referring doctor is:

☐ A neurologist *(**highly encouraged**)* ☐ A general practitioner ☐ Other: ....

French National FacioScapuloHumeral Muscular Dystrophy Patient Registry – *SELF-REPORT QUESTIONNAIRE*

Last name *(first letter)*: ___ First name *(first letter)*: ___

MANDATORY QUESTIONS

4. Have you been diagnosed with a type 1 FacioScapuloHumeral Muscular Dystrophy (FSHD1)?

☐ **Yes (*Go to question #5*)**

☐ * No

☐ * I don't know

> ***** If you answer "No" or "I don't know", stop the questionnaire and refer to your doctor to better specify your clinical diagnosis.***

5. Have you had a genetic test for FacioScapuloHumeral Muscular dystrophy type 1 (FSHD1)?

☐ **Yes, and I have received the result of the genetic test (*Go to question #6*)**

☐ ** Yes, but I am waiting for the result

☐ ** No, I never had a genetic test for FSHD

☐ ** I don't know

> ***** If you answer "No", "I don't know" or if you are waiting for your genetic results, stop the questionnaire and ask your referring doctor about your genetic diagnosis.***

6. If you have received the result of the genetic test, please specify if the test:

☐ **confirmed** the diagnosis of FSHD (***Go to question #7***) ☐ **DID NOT confirm** the diagnosis of FSHD (***Stop the questionnaire***)

7. If your genetic test confirmed the diagnosis of FSHD1, please specify: ***(to answer this question, you have to read your genetic result, see instruction for more information)***

- Year of genetic diagnosis: ___ ___ ___ ___
- Name of genetic laboratory: ☐ MARSEILLE ☐ PARIS ☐ Other, *specify* ....
- Genetic test result (***optional***): 4q D4Z4 repeat number? ___ ___ **or** EcoRI / BnI fragment length? .... kb ☐ Mosaic

8. As shown in picture on the right, are you able to (*select only one answer*):

1 ☐ Raise arms sideways overhead

2 ☐ Raise arms sideways up to, but not above, shoulder height

3 ☐ You are not able to raise arms

4 ☐ I don't know

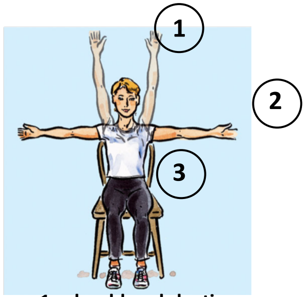

Figure 1 : shoulder abduction

9. Are you able to walk without support at all times (cane, walker, wheelchair…)?

☐ **Yes*** ☐ No ☐ I don't know **(*** ***If Yes, go to question #11*)**

Last name ***(first letter):*** ___ **First name** ***(first letter):*** ___

MANDATORY QUESTIONS

10. If you walk with an aid, please specify the type of aid you use *(several choices are possible)*:

☐ Cane ☐ Walker ☐ Ankle orthosis ☐ Manual wheelchair ☐ Electric wheelchair

☐ Triscooter ☐ Other, *specify*: ....

11. Have you already taken part in a clinic trial/study on FSHD?

☐ Yes ☐ No ☐ I don't know

12. Do you want to be informed about future clinical trials/studies on FSHD?

☐ Yes ☐ No ☐ I don't know

HIGHLY ENCOURAGED QUESTIONS – *You will be included in the registry even if you do not answer all of these questions, but please answer as many of them as you can.*

13. Please indicate your height and weight

Height ___ ___ ___ cm Weight ___ ___ ___ kg

14. Please specify if you are

☐ Right-handed ☐ Left-handed ☐ Ambidextrous

15. Please indicate your marital status

☐ Single ☐ Domestic partnership ☐ Civil union (PACS) ☐ Divorced ☐ Married ☐ Widowed

16. What is your employment status?

☐ Employed ☐ Student ☐ Retired ☐ Unemployed ☐ Never employed ☐ Termination of employment → *due to FSHD:* ☐ *Yes* ☐ *No*

17. What is the highest degree or level of school you achieved?

☐ No diploma ☐ Primary School certificate ☐ Vocational school (BEP / CAP) ☐ 10th Grade ☐ 11th Grade ☐ 12th Grade ☐ High-school diploma (baccalauréat)
☐ Higher education (Baccalauréat +) .... ☐ Other: ....

Last name *(first letter)*: ___ **First name** *(first letter)*: ___

HIGHLY ENCOURAGED QUESTIONS

18. To your knowledge, is any member of your family suffering from FSHD?

☐ Yes ☐ No* ☐ I don't know* (*Go to question #20)

19. If yes, please specify who else is affected in your family? ***(several choices are possible)***

☐ Mother ☐ Father ☐ Brother(s) ☐ Sister(s) ☐ Son ☐ Daughter ☐ Other(s): ....

20. How old were you when the first symptom of FSHD appeared?

___ ___ **years old**

21. What was your FIRST symptom of FSHD? ***(PLEASE SELECT ONLY ONE ANSWER)***

☐ I don't have any symptom

☐ Facial weakness (difficulty closing your eyes, whistling and/or drinking from a straw)

☐ Upper limbs proximal weakness (difficulty raising arms)

☐ Upper limbs distal weakness (difficulty using your hands)

☐ Lower limbs proximal weakness (difficulty climbing stairs)

☐ Lower limbs distal weakness (difficulty walking on your heels)

☐ Others, *briefly describe your first symptom*: ....

SOME QUESTIONS ABOUT YOUR FACIAL WEAKNESS

22. Are your eyes often irritated and/or dry?

☐ Yes ☐ No ☐ I don't know

23. Do you sleep with your eyes open?

☐ Yes ☐ No ☐ I don't know

24. Do you have difficulties to close your eyes?

☐ Yes ☐ No ☐ I don't know

25. Are you able to whistle and/or pucker your lips?

☐ Yes ☐ No ☐ I don't know

<u>Last name</u> ***(first letter):*** ___ <u>First name</u> ***(first letter):*** ___

HIGHLY ENCOURAGED QUESTIONS

SOME QUESTIONS ABOUT YOUR UPPER LIMB WEAKNESS

26. Please encircle <u>ONE</u> of the following answers *(<u>SELECT ONLY ONE ANSWER</u>)*:

1. I can raise my arms overhead <u>without bending my elbows</u> WITHOUT HELP (***<u>see figure 1</u>***) →
2. I can raise my arms overhead <u>only by bending my elbows</u> (reducing the circumference of the movement)
3. I can't raise my arms overhead, but I can raise a glass full of water to my mouth (using one or both hands)
4. I can raise my hands to touch my lips, but I can't raise a glass full of water to my mouth
5. I can't raise my hands to touch my lips, but I can lift a pencil from a table
6. I can't use my hands

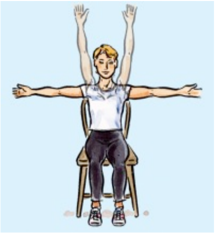

Figure 1: Shoulder abduction

27. Do you have scapular winging *(<u>see figure 2</u>)*?

☐ Yes ☐ No ☐ I don't know

28. Have you had a surgery to fix your winged shoulder blade(s)?

☐ Yes ☐ No ☐ I don't know

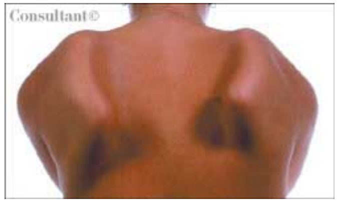

Figure 2 : Scapular winging

SOME QUESTIONS ABOUT YOUR LOWER LIMB WEAKNESS

29. Please encircle <u>ONE</u> of the following answers *(<u>SELECT ONLY ONE ANSWER</u>)*:

1. I walk and climb stairs without support
2. I walk and climb stairs using the handrail
3. I walk and climb stairs very slowly using the hand rail (more than 12 seconds for 4 steps)
4. I can walk and get up from a chair without support, but I can't climb stairs
5. I can walk without support, but I can't get up from a chair without support and I can't climb the stairs
6. I can walk only with support or with an ankle-foot orthosis
7. I can walk only with a cane or a walker
8. I can't walk, but I can stand up with support
9. I am confined to wheelchair
10. I am confined to bed

Last name ***(first letter):*** ___ **First name** ***(first letter):*** ___

HIGHLY ENCOURAGED QUESTIONS

30. Do you have difficulties walking on your heels? *(see figure 3)*

☐ Yes ☐ No ☐ I don't know ☐ Not applicable *(wheelchair or confined to bed)*

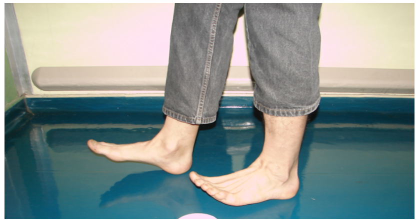

Figure 3 : Walking on heels

31. Do you have difficulties getting out of bed without using your arms?

☐ Yes ☐ No ☐ I don't know

32. Is one of your arms or legs more affected than the other?

☐ Yes ☐ No ☐ I don't know

OTHER CONDITIONS

33. Have you been diagnosed with a cardiac condition?

☐ **Yes** ☐ No ☐ I don't know

→ ***If yes,*** *specify: ....*

34. Have you been diagnosed with a respiratory condition?

☐ **Yes** ☐ No ☐ I don't know

→ ***If yes,*** *specify: ....*

→ *Do you use a respiratory assist device?* ☐ *Yes* ☐ *No*

35. Have you been diagnosed with a hearing disorder?

☐ **Yes** ☐ No ☐ I don't know → ***If yes,*** *do you use a hearing aid?* ☐ *Yes* ☐ *No*

36. Have you been diagnosed with a swallowing disorder?

☐ Yes ☐ No ☐ I don't know

37. Have you been diagnosed with an ocular condition?

☐ **Yes** ☐ No ☐ I don't know → ***If yes,*** *specify the diagnosis: ....*

38. Have you had eye surgery?

☐ **Yes** ☐ No ☐ I don't know → ***If yes,*** *specify the medical reason of this surgery: ....*

Last name ***(first letter):*** ___ **First name** ***(first letter):*** ___

HIGHLY ENCOURAGED QUESTIONS

39. Have you ever been diagnosed with one of the following endocrine disorders?

☐ **Yes** ☐ No ☐ I don't know

→ ***If yes,*** *specify:* ☐ Diabetes ☐ Thyroid disease ☐ Other ……………………………….

40. Have you ever been diagnosed with one of the following lipid disorders?

☐ Yes ☐ No ☐ I don't know

→ ***If yes,*** *specify:* ☐ Triglycerides ☐ Cholesterol

→ ***If yes,*** *specify if you take any medication to lower cholesterol and/or triglycerides:*

☐ None ☐ Statin ☐ Fibrate ☐ Other ……………………………….

41. Check the boxes corresponding to symptoms you have already shown: ***several choices are possible***

☐ Palpitations
☐ Syncope, fainting *(loss of consciousness)*
☐ Dizziness *(feeling faint but without loss of consciousness)*
☐ Retrosternal pain *(chest pain)*

☐ Dyspnea on exertion *(difficulty breathing on exertion)*
☐ Dyspnea at rest *(difficulty breathing at rest)*
☐ Orthopnea *(difficulty breathing when lying down)*
☐ Daytime sleepiness
☐ Morning headaches

Other, specify:

……………………………………………………………………………………………

☐ No symptoms

☐ Dysphagia *(difficulty swallowing, food or liquid getting stuck during meals)*
☐ Swallowing the wrong way (*food or liquid going "down the wrong pipe", into the superior airways, causing coughing, choking, nasal regurgitations*)
☐ Weight loss

☐ Flashes
☐ Floaters (*vision of spots, dots, wavy lines, cloud-like shapes, cobwebs*)
☐ Blurred vision; acute vision loss
☐ Red eyes
☐ Pain in the eyes
☐ Feeling of something in your eye (watery eyes)

☐ Decrease of hearing

Last name ***(first letter):*** ___ **First name** ***(first letter):*** ___

HIGHLY ENCOURAGED QUESTIONS

SOME QUESTIONS ABOUT YOUR PAIN ASSESSMENT

42. Do you have any joint or muscle pain?

☐ Yes ☐ No ☐ I don't know

43. According to the intensity of your daily pain, please draw a vertical line on the horizontal line below:

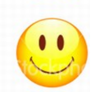 **No pain** ____ **Extremely painful** 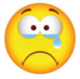

44. In which area(s) do you localize your pain? (several choices are possible)

☐ No pain ☐ Neck/upper back ☐ Lower back/hips ☐ shoulders/upper arms

☐ Knees/thighs ☐ Elbows ☐ Ankles/lower legs ☐ Other: ……………………………………………………………………………………

SOME QUESTIONS ABOUT THE FRENCH NATIONAL FSHD PATIENT REGISTRY

45. Would you agree that a neuromuscular neurologist fill a *clinical evaluation form to assess your condition?**

☐ Yes ☐ No ☐ I don't know

46. Has your referring neurologist already filled out the *clinical evaluation form?**

☐ Yes ☐ No ☐ I don't know ☐ I don't have a referring neurologist

* **The clinical evaluation form,** has to be filled out by a neurologist specialized in neuromuscular disorders. This form will allow a detailed evaluation of your medical status as well as collecting important information on the history of your disease. The results of this evaluation will be sent to your general practitioner and will be part of your profile in the French National Registry on FacioScapuloHumeral Muscular Dystrophy (please read the information notes for more details or go to the website http://www.fshd.fr).

END OF THE QUESTIONNAIRE!

Thank you for taking the time to complete it and don't forget to update your information by filling out a new questionnaire EVERY TWO YEARS.
