# Supplementary material for: The French National Registry of patients with Facioscapulohumeral muscular dystrophy
Source: Orphanet J Rare Dis. 2018 Dec 4;13:218. doi: 10.1186/s13023-018-0960-x (PMC6280451; doi:10.1186/s13023-018-0960-x)
Supplement: Supplementary file 3 — Figure S3. French National FSHD registry - INCLUSION FORM – FSHD LIKE (PDF 473 kb) [file 13023_2018_960_MOESM3_ESM.pdf]

French National Registry on FSHD

CLINICAL EVALUATION FORM "FSHD with no D4Z4 contraction"

- **Last Name** *(initial)* |__| **First Name** *(initial)* |__|
- **Birth date** *(month/year)*: _____/_______ - **Gender:** F ❑ M ❑
- **Primary residence:** _________________________________

Date of consult: __ __/ __ __/__ __ __ __

☐ **Form #1 (INCLUSION)**

☐ **Follow-up form #**|____| → **PIN:** _______

Name of the physician specialized in neuromuscular disorders recruiting the patient: ____<br>**Center:** ____	Signature	Office stamps of physician (including name)

A. CIRCUMSTANCES AND ELEMENTS OF DIAGNOSIS

To be completed at inclusion only

1. Year of clinical diagnosis __ __ __ __ **2. Age at first symptoms** __ __ years old

3. First symptom according to patient

☐ Asymptomatic patient ☐ Facial involvement
☐ Proximal upper limb involvement ☐ Proximal lower limb involvement
☐ Distal upper limb involvement ☐ Distal lower limb involvement
☐ Other → *if other, specify:* _______________________________________

4. Family history: *affected family members (several answers possible)*

☐ None ☐ Mother ☐ Father ☐ Brothers ☐ Sisters ☐ Others: _______________________________

→ **GENEALOGICAL TREE:** ○ = *healthy woman;* ☐ = *healthy man;* ● = *affected woman;* ■ = *affected man*

5. Molecular diagnostic of FSHD1

a. **Laboratory:** ☐ Paris ☐ Marseille
☐ Other: ______________________

b. **Analysis year:** ___ ___ ___ ___

c. **4q35/ D4Z4: number of repeats:** |__|__| *or* **ECORI fragment length:** ________ kb (from 10 to 38)

d. **Mutation in SMCHD1 gene:** ☐ **Yes** ☐ No
☐ Not done

e. **Methylation percentage:** "Fsel % methylation" ___________ % ☐ Not done

B. DIAGNOSIS COMPLEMENT *To be completed at inclusion AND follow-up*

☐ Data unchanged since last form

1. **Other genetic tests: check the genetic tests that have been performed on your patient** *(several choices are possible):*

2.

☐ Calpain-3	→	Results:	○ negative	○ pending	○ not done
☐ Dysferlin	→	Results:	○ negative	○ pending	○ not done
☐ VCP	→	Results:	○ negative	○ pending	○ not done
☐ FHL1	→	Results:	○ negative	○ pending	○ not done
☐ Others → *Specify:* ____________	→	Results:	○ negative	○ pending	○ not done
____________	→	Results:	○ negative	○ pending	○ not done
____________	→	Results:	○ negative	○ pending	○ not done

3. **Other blood tests: check blood tests that have been performed on your patient** *(several choices are possible):*

4. ☐ CPT-II in leukocytes → Results: ◯ negative ◯ pending ◯ not done

☐ Dried blood spot GAA testing (*Pompe disease*) → Results: ◯ negative ◯ pending ◯ not done

☐ GAA in lymphocytes (*Pompe disease*) → Results: ◯ negative ◯ pending ◯ not done

☐ Lowest CPK values: Year: ___ ___ ___ ___ ___ ___ ___ x normal value

5. **EMG** ☐ not done ☐ **done** → Result: ☐ Normal ☐ Myogenic ☐ Neurogenic

6. **Muscle biopsy:** ☐ not done ☐ **done** → Date of the muscle biopsy: ____/____/_____

Result: ☐ Normal (go to question #b) *or* ☐ Abnormal, *specify:*

a. Histology / Histochemistry: *if the biopsy is abnormal, specify (several choices are possible):*

- Dystrophy → ☐ no ☐ yes
- Necrosis → ☐ no ☐ yes
- Inflammation → ☐ no ☐ yes
- Selective fiber atrophy → ☐ no ☐ yes
- Fiber-type grouping → ☐ no ☐ yes
- Vacuoles → ☐ no ☐ yes
- Inclusions → ☐ no ☐ yes
- Ragged red fibers or COX-neg. fibers → ☐ no ☐ yes, *specify number:* ____ ____
- Others →*Specify:* _

b. Immunohistochemistry: *check the immunohistochemical analyzes done on the muscle biopsy (several choices are possible):*

☐ DYS 1,2,3 ☐ Sarcoglycans ☐ Caveolins ☐ Dysferlin ☐ α-/β dystroglycans ☐ Merosin

☐ Other, *specify:* _

c. Western blot: *check all western blots done on the muscle biopsy (several choices are possible):*

☐ DYS 1,2,3 ☐ Sarcoglycans ☐ Calpain-3 ☐ Dysferlin ☐ α-/β dystroglycans

☐ Other, *specify:* _

d. Additional tests: *check all additional tests done on the muscle biopsy (several choices are possible):*

☐ Calpain-3 autocatalytic activity

☐ Dosage of mitochondrial respiratory chain enzymes

☐ Activity of glycolysis and glycogenosis enzymes

☐ Other, *specify:* _

5. **Muscle MRI**

☐ not done ☐ **done**, *specify:* ☐ Upper limbs ☐ Lower limbs ☐ Full body

C. FOLLOW-UP DATA *To be completed at inclusion AND follow-up*

☐ Data unchanged since last form

1. **Current civil status**

☐ Single ☐ Separated ☐ Domestic partnership ☐ Civil union (PACS) ☐ Married ☐ Divorced ☐ Widowed

2. **Employment status**

☐ Employed ☐ Unemployed ☐ Retired ☐ Never employed ☐ Termination of employment, *check if due to FSHD:* ☐

☐ Pupil / Student → **Education level:** _

→ **Education environment:** ☐ Normal (no help) ☐ Normal with help (support personnel, adjusted schedules) ☐ Specialized

3. **Biometric data**

Height ___ ___ ___ **cm** / Weight ___ ___ ___ **kg**

4. **Handedness:**

☐ Right-handed ☐ Left-handed ☐ Ambidextrous

D. MUSCULAR AND FUNCTIONAL EVALUATIONS *To be completed at inclusion AND follow-up*

☐ Data unchanged since last form

1. Facial involvement	Un-affected	Affected	Un-certain
Wrinkle forehead			
Close eyes			
Smile			
Pucker lips (mimic a kiss)			
Show teeth (platysma)			

2. Tongue atrophy/weakness: ☐ No ☐ Yes

3. Weakness of the scapula fixator muscles:

Scapular winging: ☐ No ☐ Yes

4. Axial involvement: ☐ No ☐ Yes

5. Ability to walk: ☐ No ☐ Yes

6. Functional autonomy:

- **Getting out of bed:** ☐ without using arms ☐ without using arms ☐ with the help of someone
- **Getting up from a chair:** ☐ without using arms ☐ without using arms ☐ with the help of someone

7. Manual muscle testing: 0-5

FACIAL INVOLVEMENT	R	L
Orbicularis oculi	|____|	|____|
Orbicularis oris	|____|	|____|
**AXIAL INVOLVEMENT**		
Neck flexor muscles	|____|	
Neck extensor muscles	|____|	
Trunk flexor muscles	|____|	
Trunk extensor muscles	|____|	
**UPPER LIMBS**	**R**	**L**
Shoulder antepuslion	|____|	|____|
Right scapular retropulsion (fixator)	|____|	|____|
Shoulder Abduction: overall	|____|	|____|
Shoulder abduction: deltoid muscle *(the scapula is held manually)*	|____|	|____|
Forearm flexion (biceps)	|____|	|____|
Forearm extension (triceps)	|____|	|____|
Wrist flexion	|____|	|____|
Fingers flexion	|____|	|____|
Fingers extension	|____|	|____|
**LOWER LIMBS**	**R**	**L**
Hip flexion (ilio-psoas)	|____|	|____|
Leg extension (quadriceps)	|____|	|____|
Leg flexion (hamstrings)	|____|	|____|
Foot dorsiflexion (tibialis anterior)	|____|	|____|
Plantar flexion (triceps sural)	|____|	|____|

Comments: _ **Asymmetry:** ☐ No ☐ Yes

D. MUSCULAR AND FUNCTIONAL EVALUATIONS
(continued)

8. Beevor's sign: *Movement of the navel towards the neck on flexing the neck (weakness of the lower abdominal muscles)*

☐ Untested ☐ Absent ☐ Present: direction ↑ _______ cm / direction ↓ _______ cm

9. Functional tests

**A. UPPER LIMBS**		
**Active elevation of arms** *(degrees)*	**L** ___ ___ ___° **R** ___ ___ ___° ☐ Not done	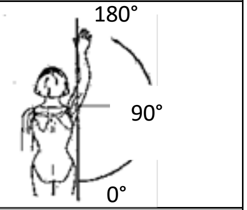
**Brooke scale**	___ / 6 (*see appendix #1*) ☐ Not done	
**B. LOWER LIMBS**		
**Timed 10-meter walk test**	___ ___ sec. ☐ NA* ☐ Not done	☐ With assistance → *Specify:* ___________<br>☐ No assistance
**Four square step test**	___ ___ ___ sec. ☐ NA* ☐ Not done	☐ With assistance → *Specify:* _________<br>☐ No assistance
**Vignos scale**	___ / 10 (*see appendix #1*) ☐ Not done	
**C. CLINICAL SEVERITY SCORE**		
	___ ___ / 10 (*see appendix #2*) ☐ Not done	

10. Reduced joint flexibility: ☐ No ☐ Yes →*Specify*: ☐ Shoulders ☐ Elbows ☐ Wrists ☐ Fingers ☐ Hips ☐ Knees ☐ Ankles ☐ Other, *specify:* __________

11. Joint or muscle pain: ☐ No ☐ Yes

If yes, specify:

a. **Date of onset** *(month / year)*: _____ /________

b. **Visual Analog Scale (VAS) on the day of consult: place a thin line perpendicular to the VAS line** ***(done by the patient)***

No pain ____ Extremely painful

c. **Pain management:** ☐ **None** ☐ **Stretching** ☐ **Massage**

☐ **Drugs** → ***specify***: __________________ ☐ **Others** → ***specify***: __________________

E. THERAPEUTIC CARE
☐ Data unchanged since last form

1. Non-drug treatment:

☐ None ☐ Physical therapy → *number of sessions /week:* __ __

☐ Orthophony → *number of sessions / week:* __ __ ☐ *Scapular fixation surgery* → ○ Right ○ Left

2. Assistive device

☐ None ☐ Cane → ☐ simple ☐ crutch ☐ tripod

☐ Walker

☐ Manual wheelchair → frequency of use: ☐ occasionally ☐ regularly ☐ exclusively

☐ Electric wheelchair → frequency of use: ☐ occasionally ☐ regularly ☐ exclusively

☐ Other, *specify*: ____

F. CARDIAC INVOLVEMENT *To be completed at inclusion AND follow-up*

☐ Data unchanged since last form ☐ Not assessed ☐ ABSENCE ☐ **PRESENCE ***

***please complete below**

1. **Age at onset:** __ __ → ☐ Patient interview data or ☐ Follow-up data
2. **Symptoms:** ☐ Palpitations ☐ Syncopes, fainting ☐ Vertigo, dizziness ☐ Retrosternal pain
   ☐ No symptom but but abnormal exam ☐ Other, *specify*_ _ _ _ _ _ _ _ _ _ _ _ _ _ _ _ _ _ _ _
3. **Cardiac exams** *(if done, specify for each exam **the month and the year**)*:

**ECG**	☐ **Done**___/________<br>☐ Not done	☐ **No anomaly** ☐ Conduction block ☐ Arrhythmia<br>☐ Others, *specify*: _
**Holter test**	☐ **Done**___/________<br>☐ Not done	☐ **No anomaly** ☐ Conduction block ☐ Arrhythmia<br>☐ Others, *specify*: _
**Echo-cardiography**	☐ **Done**___/________<br>☐ Not done	- **Left Ventricular Ejection Fraction:** ___ ___ %<br>- **Cardiomyopathy:**<br>☐ No ☐ **Yes** → ☐ Dilated ☐ Hypertrophic ☐ Other: _ _ _ _ _ _ _<br>- **Other,** *specify*: _

4. **Diagnosis retained:** _

G. RESPIRATORY INVOLVEMENT *To be completed at inclusion AND follow-up*

☐ Data unchanged since last form ☐ Not assessed ☐ ABSENCE ☐ **PRESENCE ***

***please complete below**

1. **Age at onset** ___ ___ → ☐ Patient interview data or ☐ Follow-up data
2. **Symptoms:** ☐ Dyspnea on exertion ☐ Dyspnea at rest ☐ Orthopnea
   ☐ Daytime sleepiness ☐ Morning headaches ☐ Others, *specify:* _ _ _ _ _ _ _ _ _ _ _ _ _
3. **Concomitant skeletal anomalies:**
   ☐ Spine deformation ☐ Pectus excavatum ☐ Other, *specify:* _ _ _ _ _ _ _ _ _ _ _ _ _ ☐ None
4. **Exams** *(if done, specify for each exam **the month and the year**):*

**PFT**	☐ **Done**___/________<br>☐ Not done	FVC sitting ___ ___ ___% FVC supine ___ ___ ___ **%**
**Blood gases at rest**	☐ **Done**___/________<br>☐ Not Done	PO_2 __ __ __ mmHg PCO_2 __ __ __ mmHg Sat __ __ __ %
**Polysomnography**	☐ **Done**___/________<br>☐ Not Done	**Type of apnea:** ☐ Obstructive ☐ Central ☐ Mixed<br>Apnea index: |______|per hour

5. **Assisted ventilation:** ☐ No ☐ **Yes** → ☐ **Invasive (tracheostomy)** ☐ Non-invasive, nasal mask
   → ○ **Permanent** ○ **Intermittent, as required** ○ **Intermittent, nocturnal**

H. OCULAR INVOLVEMENT *To be completed at inclusion AND follow-up*

☐ Data unchanged since last form ☐ Not assessed ☐ ABSENCE ☐ **PRESENCE ***

***please complete below**

1. **Age at onset:** ___ ___ → ☐ Patient interview data or ☐ Follow-up data
2. **Diagnosis:** ☐ Vascular retinopathy ☐ Retinal Detachment ☐ Coats' disease ☐ Corneal ulcer
   ☐ Others, *specify*: ____
3. **Surgery:** ☐ No ☐ Yes → *if yes, reason of surgery*: ________________________________

I. AUDITORY INVOLVEMENT *To be completed at inclusion AND follow-up*

☐ Data unchanged since last form ☐ Not assessed ☐ ABSENCE ☐ **PRESENCE***

*** please complete below**

Year of last audiogram: ___ ___ ___ ___ ☐ ***Not done***

1. **Age at onset:** ___ ___ → ☐ Patient interview data or ☐ Follow-up data
2. **Hearing aid:** ☐ No ☐ Yes

J. GASTRO-INTESTINAL INVOLVEMENT *To be completed at inclusion AND follow-up*

☐ Data unchanged since last form ☐ Not assessed ☐ ABSENCE ☐ **PRESENCE ***

***please complete below**

1. **Age at onset:** ___ ___ → ☐ Patient interview data or ☐ Follow-up data
2. **Symptoms:** ☐ Dysphagia ☐ Pulmonary aspiration → *specify number of pulmonary aspirations*: ______ / week
   ☐ Other, *specify*: ____
3. **Endoscopic evaluation of swallowing:** ☐ Not done ☐ **done:** Dysphagia detected ? : ☐ No ☐ Yes

K. METABOLIC INVOLVEMENT *To be completed at inclusion AND follow-up*

☐ Data unchanged since last form ☐ Not assessed ☐ ABSENCE ☐ **PRESENCE ***

***please complete below**

1. **Age at onset:** ___ ___ → ☐ Patient interview data or ☐ Follow-up data
2. **Diagnosis:** ☐ Hypercholesterolemia ☐ Hypertriglyceridemia ☐ Other, *specify:* ________________
3. **Lipid-lowering treatment:** ☐ No ☐ Yes → *specify name(s) and length of treatment(s):*
   ____

L. ENDOCRINE INVOLVEMENT

To be completed at inclusion AND follow-up

☐ Data unchanged since last form ☐ Not assessed ☐ ABSENCE ☐ **PRESENCE ***

*please complete below

1. **Age at onset:** ___ ___ → ☐ Patient interview data or ☐ Follow-up data
2. **Type of disease:** ☐ Thyroid disorder ☐ Diabetis ☐ Other, *specify*: _ _ _ _ _ _ _ _ _ _ _ _
3. **Hormone therapy:** ☐ No ☐ Yes → *specify name(s) and length of treatment(s):*

   _
4. **Sexual hormone therapy:** ☐ No ☐ Yes → *specify name(s) and length of treatment(s):*

   _

FOR ALL FEMALE SUBJECTS

☐ Pre-puberty ☐ Puberty ☐ Pre-menopause ☐ Menopause → *Age at menopause:* ___ ___

- **Contraceptive treatment:** ☐ None ☐ Oral contraceptive ☐ Other, *specify*: _ _ _ _ _ _ _ _ _ _ _ _ _ _ _ _ _

- **Pregnancy:** Number of pregnancies (gestity): ___ ___ Number of children: ___ ___

M. TYPICAL AND ATYPICAL CHARACTERISTICS OF FSHD

To be completed at inclusion AND follow-up

☐	**TYPICAL:** at least 2 criteria in favor of a FSHD1 diagnosis AND 0 atypia criterion
☐	**WITH ATYPIA:** at least 1 atypia criterion

Check matching criteria

	**CRITERIA IN FAVOR OF FSHD1**
☐	Asymetry
☐	Facial involvement (orbicularis oculi or orbicularis oris)
☐	Involvement of scapular fixator or tibialis anterior
	**ATYPIA CRITERIA**
☐	Ptosis, ophthalmoplegia or diplopia
☐	Cardiological or respiratory involvement in the foreground OR SNC involvement
☐	Predominant involvement at the proximal lower limb or distal upper limb level
	**+**
☐	EMG suggesting a neurogenic motor neuron disease or myotonia
☐	Muscle biopsy atypical or suggesting a muscle disorder
☐	Genetic diagnosis of another neuromuscular disorder confirmed
☐	Others: _

APPENDIX 1

BROOKE AND VIGNOS SCALES

UPPER LIMBS (Arms and shoulders)	LOWER LIMBS
**1 –** Starting with arms at the sides, the patient can abduct the arms (kept extended) in a full circle until they touch above the head.	**1 –** Walks and climbs stairs without assistance.
**2 –** Can raise arms above head only with elbows flexed or by using accessory muscles.	**2 -** Walks and climbs stair with the aid of the railing.
**3 -** Can raise hands to the mouth but cannot raise an 8-oz glass of water to the mouth (using both hands if necessary).	**3 –** Walks and climbs stairs slowly with the aid of the railing (over 12 seconds to climb four stairs).
**4 –** Can raise hands to the mouth but cannot raise an 8-oz glass of water to the mouth.	**4 –** Walks unassisted and rises from a chair but cannot climb stairs.
**5 -** Cannot raise hands to the mouth but can use hands to hold a pen or pick up pennies from the table.	**5 –** Walks unassisted but cannot rise from a chair or climb stairs.
**6 –** Cannot raise hands to the mouth and has no useful function of hands.	**6 –** Walks only with assistance or walks independently with long leg braces.
	**7 –**Walks with long leg braces but requires assistance for balance.
	**8 –** Stands with long leg braces but unable to walk even with assistance
	**9 –** Wheelchair-bound.
	**10 –** Bedridden.

APPENDIX 2

CLINICAL SEVERITY SCORE

0 = No sign of muscle weakness.

1 = Facial weakness only.

2 = Mild scapular involvement without limitation of arm abduction; no awareness of disease symptoms is possible.

3 = Moderate involvement of scapular and arm muscles or both (arm abduction >60° and strength ≥3 in arm muscles); no involvement of pelvic and leg muscles.

4 = Severe scapular involvement (arm abduction <60°on at least one side); strength <3 in at least one muscular district of the arms; no involvement of pelvic and leg muscles.

5 = Tibioperoneal weakness; no weakness of pelvic and proximal leg muscles.

6 = Mild weakness of pelvic and proximal leg muscles or both (strength ≥4 in all these muscles); able to stand up from a chair without support.

7 = Moderate weakness of pelvic and proximal leg muscles or both (strength ≥3 in all these muscles); able to stand up from a chair with monolateral support.

8 = Severe weakness of pelvic and proximal leg muscles or both (strength <3 in at least one of these muscles); able to stand up from a chair with double support; unable to climb stairs; able to walk unaided.

9 = Unable to stand up from a chair; walking limited to several steps with support; may use wheelchair for most activities.

10 = Wheelchair-bound.
