# Supplementary material for: The French National Registry of patients with Facioscapulohumeral muscular dystrophy
Source: Orphanet J Rare Dis. 2018 Dec 4;13:218. doi: 10.1186/s13023-018-0960-x (PMC6280451; doi:10.1186/s13023-018-0960-x)
Supplement: Supplementary file 4 — Table S1. Pre-filling rules for the self-report and the clinical evaluation questionnaires. (PDF 59 kb) [file 13023_2018_960_MOESM4_ESM.pdf]

<u>Pre-filling</u>

Self-report questionnaire

Condition	Pre-filling
Physician's name is filled and already known	Autocompletion of other fields
Age of onset is set to "Never"	*First symptom* is set to "Asymptomatic"
Age of onset goes from "Never" to something else	*First symptom* is reset
First symptom is set to "Asymptomatic"	*Age of onset* is set to "Never"
First symptom goes from "Asymptomatic" to something else	*Age of onset* is reset
Muscular and joint pain is set to "No"	*Average daily pain* is set to "0.0"
	Pain area is set to "No pain"
Muscular and joint pain goes from "No" to "Yes"	*Average daily pain* is reset
	Pain area is reset
Average daily pain is set to "0.0"	*Muscular and joint pain* is set to "No"
	Pain area is set to "No pain"
Average daily pain goes from "0.0" to something else	*Muscular and joint pain* is reset
	Pain area is reset
Pain area is set to "No pain"	If other checkboxes were set for *Pain area*, they are reset
	Muscular and joint pain is set to "No"
	Average daily pain is set to "0.0"
Pain area goes from "No pain" to something else	*Muscular and joint pain* is set to "Yes"
	If *Average daily pain* was set to "0.0", it is reset

Clinical evaluation questionnaire

Condition	Pre-filling
Age of onset is set to "Never"	*First symptom* is set to "Asymptomatic"
Age of onset goes from "Never" to something else	*First symptom* is reset
First symptom is set to "Asymptomatic"	*Age of onset* is set to "Never"
First symptom goes from "Asymptomatic" to something else	*Age of onset* is reset
If one of the four items of *Manual muscular testing: Axial involvement* is strictly inferior to "5"	*Axial involvement* is set to "Yes" and an alert is displayed to inform the curator
If one of the two items of *Manual muscular testing: Scapula retropulsion* is strictly	*Scapula stabilizer muscle weakness* is set to "Yes" and an alert is displayed to inform the

inferior to “5”	curator
If one of the 16 values of *Manual muscular testing: Right* is different from *Manual muscular testing: Left*	*Asymmetry* is set to “Yes”
If all 16 values of *Manual muscular testing: Right* are the same than *Manual muscular testing: Left*	*Asymmetry* is set to “No”
Ambulation is set to “No”	*10mt walking test* is set to “Not applicable” and an alert is displayed to inform the curator
	Four-step test is set to “Not applicable” and an alert is displayed to inform the curator
Number of pregnancies is set to “0”	*Number of children* is set to “0” and can’t be modified
Number of pregnancies goes from “0” to something else	*Number of children* is reset
