# Supplementary material for: The French National Registry of patients with Facioscapulohumeral muscular dystrophy
Source: Orphanet J Rare Dis. 2018 Dec 4;13:218. doi: 10.1186/s13023-018-0960-x (PMC6280451; doi:10.1186/s13023-018-0960-x)
Supplement: Supplementary file 5 — Table S2. Conflicts identification rules for the clinical evaluation questionnaire. (PDF 54 kb) [file 13023_2018_960_MOESM5_ESM.pdf]

Conflicts

Clinical evaluation questionnaire

Action	Alert displayed if:	Reset field
Axial involvement is set to "No"	One of the four items of *Manual muscular testing: Axial involvement* is strictly inferior to "5"	*Axial involvement*
Scapula stabilizer muscle weakness is set to "No"	One of the two items of *Manual muscular testing: Scapula retropulsion* is strictly inferior to "5"	*Scapula stabilizer muscle weakness*
Asymmetry is set to "Yes"	All 16 values of *Manual muscular testing: Right* are the same than *Manual muscular testing: Left*	*Asymmetry* is set to "No"
Asymmetry is set to "No"	One of the 16 values of *Manual muscular testing: Right* is different from *Manual muscular testing: Left*	*Asymmetry* is set to "Yes"
Ambulation is set to "Yes"	*Vignos scale* equals to "9" or "10"	*Vignos scale*
Vignos scale is set to "9" or "10"	*Ambulation* equals "Yes"	*Ambulation*
10mt walking test is set to something else than "Not applicable"	*Ambulation* equals "No"	*10mt walking test*
Four-step test is set to something else than "Not applicable"		*Four-step test*
Assisted-mobility devices is set to "exclusive use" for "manual wheelchair" and/or "electric wheelchair"	An alert is displayed to inform the curator to verify if:<br>- *Ambulation* is set to "No"<br>- *10mt walking test* is set to "Not applicable"<br>- *Four-step test* is set to "Not applicable"	
Ambulation is set to "Yes"	*Assisted-mobility devices* equals "exclusive use" for "manual wheelchair" and/or "electric wheelchair"	*Ambulation*
10mt walking test is set to something else than "Not applicable"		*10mt walking test*
Four-step test is set to something else than "Not applicable"		*Four-step test*
Vignos scale is strictly inferior to "8"		*Vignos scale*
Clinical severity score is strictly inferior to "9"		*Clinical severity score*
