# Supplementary figures and images for: The French National Registry of patients with Facioscapulohumeral muscular dystrophy
Source: Orphanet J Rare Dis. 2018 Dec 4;13:218. doi: 10.1186/s13023-018-0960-x (PMC6280451; doi:10.1186/s13023-018-0960-x)

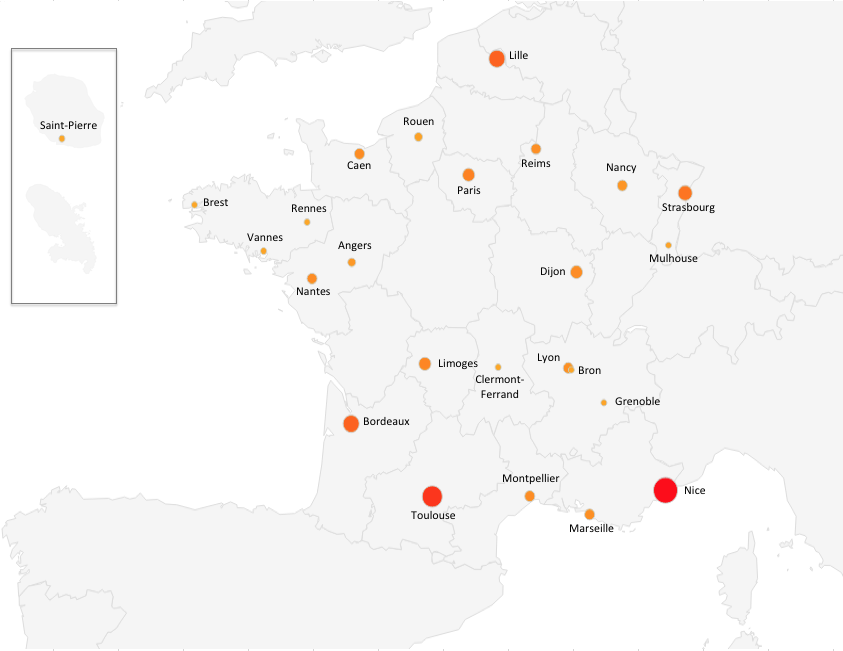

Supplement: Supplementary file 6 — Figure S4. Network of reference centers contributing to the French FSHD registry (PNG 112 kb) [file 13023_2018_960_MOESM6_ESM.png]
