# Supplementary material for: The French National Registry of patients with Facioscapulohumeral muscular dystrophy
Source: Orphanet J Rare Dis. 2018 Dec 4;13:218. doi: 10.1186/s13023-018-0960-x (PMC6280451; doi:10.1186/s13023-018-0960-x)
Supplement: Supplementary file 7 — Table S3. Mapping of clinical terms to reference systems. CTO = the Clinical Trials Ontology; CTV3 = the Read Codes Clinical Terms Version 3; HPO = the Human Phenotype Ontology; ICECI = the International Classification of External Causes of Injuries; ICF = the International Classification of Functioning, Disability and Health; LOINC = the Logical Observation Identifier Names and Codes; MEDDRA = the Medical Dictionary for Regulatory Activities; MESH = the Medical Subject Headings; NCIt = the National Cancer Institute Thesaurus; NLMVS = the NIH NLM Value Sets; OMIM = the Online Mendelian Inheritance in Man; PHENX = the PhenX Phenotypic Terms; SMASH = the SMASH Ontology; SNOMED-CT = the Systematized Nomenclature of Medicine - Clinical Terms; XCO = the Experimental Conditions Ontology. (PDF 40 kb) [file 13023_2018_960_MOESM7_ESM.pdf]

	CTO	CTV3<br>RCD	HPO	ICECI	ICF	LOINC	MEDDRA	MESH	NCIT	NLMVS	OMIM	PHENX	SMASH	SNOMEDCT	XCO
Walking		✓		✓	✓	✓	✓	✓	✓					✓	✓
Clinical trial	✓	✓				✓		✓	✓	✓				✓	
Height		✓				✓	✓	✓	✓	✓	✓	✓	✓	✓	
Weight	✓					✓	✓		✓	✓	✓	✓	✓		
Dominant side		✓				✓		✓						✓	
Other family members		V				✓			✓					✓	
Age at first symptom						✓		✓						✓	
First symptom															
Facial weakness		✓					✓		✓		✓			✓	
Dry eyes		✓					✓	✓	✓		✓	✓		✓	
Inability to fully close eyes during sleep											✓				
Unable to close eyes		✓												✓	
Able to whistle															
Atrophy of tongue		✓					✓				✓			✓	
Axial muscle weakness											✓				
Upper extremity								✓	✓					✓	
Lower extremity						✓		✓	✓					✓	
Brooke score															
Bone structure of scapula		✓												✓	
Detached scapula															
Scapula surgery															
Vignos score															
Difficulty walking on heels											✓				
Difficulty to get out of bed		✓				✓			✓					✓	
Difficulty to get out of a chair		✓							✓					✓	
Asymmetry		✓							✓		✓			✓	
Muscle and joint pain		✓				✓	✓		✓		✓			✓	
Pain severity		✓				✓	✓	✓	✓	✓				✓	
Area of pain															
Beevor's sign														✓	
Walking exercise test		✓					✓							✓	
Clinical severity scale	✓														
Joint limitation		✓									✓			✓	
Medicinal treatment		✓				✓		✓	✓					✓	
Walking assistive device		✓					✓							✓	
Other genetic test								✓	✓					✓	
Other blood test							✓		✓					✓	
EMG (Electromyography)		✓					✓		✓					✓	
Muscle biopsy		✓					✓		✓					✓	
MRI of muscle															
Heart disease		✓				✓	✓	✓	✓					✓	
ECG (Electrocardiography)		✓				✓	✓	✓	✓					✓	
ECG Holter (Holter monitoring)						✓	✓		✓						
Echocardiography		✓					✓	✓	✓					✓	
Respiratory disorder		✓					✓		✓					✓	
Skeletal abnormalities															
Observation of functional respiratory		✓												✓	

Blood gases		✓					✓	✓						✓	
Polysomnography		✓				✓	✓	✓	✓					✓	
Assisted breathing		✓					✓			✓				✓	
Eye disorder		✓					✓	✓	✓					✓	
Ophthalmic surgery						✓	✓		✓					✓	
Hearing disorder		✓					✓	✓	✓					✓	
Hearing aid		✓						✓						✓	
Gastrointestinal disease						✓	✓	✓	✓					✓	
Fiberoptic endoscopic evaluation of swallowing		✓												✓	
Metabolic disease		✓					✓	✓	✓					✓	
Lipid-lowering therapy		✓												✓	
Endocrine disorder		✓				✓	✓		✓					✓	
Hormone therapy		✓				✓	✓	✓	✓					✓	
TOTAL	3	37	0	1	1	20	28	22	32	5	11	3	2	45	1
